# Hospital and Institutional News

**Published:** 1914-11-07

**Authors:** 


### HOSPITAL AND INSTITUTIONAL NEWS.

### OUR READERS AND THE BELGIAN WOUNDED.

We publish accounts of the arrival in several places of wounded Belgian soldiers whose heroism has been the admiration of the whole world. Many of these men arrive without boots, and the make-shifts and suggestions made to supply the deficiency leave much to be desired. We should be glad to hear from the authorities of any hospital receiving wounded Belgian soldiers, and to know the number of pairs of boots they may require to equip them properly on their discharge. We believe our readers will be glad to meet the need of the brave Belgian soldiers by each subscribing the cost of at least one or possibly more pairs of boots. We hope to publish the cost per pair next week, and meanwhile we should be glad to receive offers at once. The Hospital will commence the list of Boot Subscriptions with one of fifty pairs of boots. We hope our readers and the public will support this simple recognition of these Belgian warriors. Every man and woman should gladly welcome this opportunity to record their goodwill by the gift of at least one pair. A Note on the problem at Gravesend Hospital will appear next week.

### THE PRICE OF CALAIS.

The enormous German losses due to their desperate attempt, hitherto unsuccessful, to break through the Allied line near the coast are reflected not only in the official reports of the fighting, but also in the influx of wounded which has reached London during the week. On Sunday last some fifty-seven train-loads of wounded reached the Metropolis, and the accounts which we publish elsewhere in this issue give some idea of the demands on hospital accommodation and of the efforts made to meet them. Among the latter must be noted the way in which London pay-hospitals and nursing homes are responding to the need for beds. A striking instance of the responsibility engendered by the recent fighting is seen in the fact that one responsible official of a nursing home paid a visit to the Continent, and as a result of what fell under his immediate observation, at once on his return caused additional beds to be placed at the disposal of the authorities, for, he said, "They will certainly be needed." Guy's Hospital has also extended hospitality to wounded officers, thirty of whom were received early in the week, and have been accommodated in a special ward, now fitted up into cubicles. Thus the general situation shows that what may be termed the reserve line of beds is being now drawn upon, and should this be insufficient, which we hope will not prove the case, it will be time to consider the remaining sources of accommodation, already outlined in The Hospital, by co-ordination with public and local authorities and their institutions. The price of Calais has been heavy, but there is every indication that it has not been paid in vain.

### THE WRECK OF THE HOSPITAL SHIP "ROHILLA."

The sad loss of the Government hospital ship *Rohilla*, which was wrecked on the rocks south of Whitby on Friday last week, is the first reminder that even the transport of wounded to this country is not without danger. Bound from Leith to Dunkirk to fetch wounded from France, the *Rohilla* was caught in the east-south-easterly gale, and in the very heavy seas which were running her position could only be located with difficulty from the shore. Rockets were sent up in large numbers, and though the Whitby lifeboat made two plucky journeys to the vessel, it appears that seventy-four lives have been lost out of a complement of some 220, many of whom were doctors and nurses. The attempts at rescue work, however, still went on, and the last of the survivors, numbering about fifty, were removed on Sunday last. The loss of life would probably have not been so great had not the vessel broken in two. Captain Neilson remained with his ship directing operations till the last moment. According to Admiralty announcements the official list of those saved includes the following medical men: Officers: Surgeon T. Cresswell, R.N.; Surgeon S. McBean, R.N.; Surgeon T. C. Litler Jones, R.N.V.R.; Surgeon A. W. Hird, R.N.V.R.; Surgeon L. S. Ashcroft. R.N.V.R.; Surgeon H. Leith Murray, R.N.V.R.; and J. W. Paul, dental surgeon.

### A SURVIVING DOCTOR'S EXPERIENCES.

A vivid description of the loss of the ill-fated hospital ship *Rohilla* is given by Dr. H. Leith Murray, one of the survivors, in a letter to his parents in Dundee. Dr. Murray states that a terrible gale was blowing. Articles of furniture were dashed about the cabins, and several times he was startled to wakefulness by the crashes, thinking the vessel had struck a mine. At 4.45 a.m. there was a terrific shock, and the ship quivered. Before long the lights went out, and huge waves commenced to break over the vessel. Within a very short time the wireless telegraph apparatus was washed away. "I helped the nurses to get their lifebelts on," Dr. Murray goes on, "and it was no easy task on account of the way we were dashed about. Then 'To the boat deck!' We were all soaked to the skin, and all we could see was that every boat was smashed to matchwood. When the dawn broke the ship had broken her back, and the aft end was swinging loosely with twenty men on it. Forward with us there were about eighty or ninety or perhaps a hundred. There were about 200 on board altogether. All the rocket lines fell short, and finally, when it was possible, the small lifeboat was steered amongst the rocks. Oh, the sight of it! I am sure I shan't sleep for nights."

### ALLEGED RED CROSS "PLOT" IN IRELAND.

The County Dublin Branch of the British Red Cross Society is involved in a very unfortunate affair, which may be described briefly as follc

On Friday last week there appeared in the Dublin papers a correspondence between the committee of the Dublin branch and Lady Aberdeen. This correspondence arose out of the mischievous result which followed the publication three weeks ago in *Sinn Fein* and the *Irish Worker* of a private letter, reproduced partly in facsimile, that was alleged to have been sent by Lady Aberdeen to the editor of the *Freeman's Journal*. The following sentence was contained in the communication: "I am afraid there is a bit of a plot amongst the Unionists to capture the Red Cross Society in Ireland and to run it in such a way from London and through county lieutenants and deputy lieutenants that it will be unacceptable to the Irish Volunteers' people." Suspicious of the circumstances surrounding its publication, the Dublin Red Cross authorities would have ignored it, had not their hand been forced by the excitement which was aroused. They therefore wrote on October 20 to Lady Aberdeen, remarking that she was made to appear responsible for charging a body of Irish people with using the Red Cross Society for political purposes, and requested her to disavow the authorship of the letter. "Under the circumstances," wrote Lady Aberdeen in the course of her reply, "I think it entirely unsuitable for me to discuss it in any way whatsoever." The committee of the County Dublin Branch feel that this reply is not satisfactory, and have sent this and further correspondence to the same effect to the Press. Irish Unionists express great indignation, and it remains to be seen what will be the effect on Red Cross work in Ireland.

### FREE CHOICE OF PANEL DOCTOR.

The problems of panel practice do not appear to diminish, and the latest is instructive as showing the difficulties so intimately associated with contract work. At a meeting of the West Riding Insurance Committee, held at Wakefield last week, Dr. Russell drew attention to the situation created by panel doctors who had gone on active service. After pointing out that medical men in the neighbourhood had agreed to attend the absentee doctor's patients gratuitously, he remarked that the time was now at hand when panel patients would have the opportunity of changing their doctors. Under these circumstances, therefore, he proposed that a panel patient of any doctor engaged on military service should not be allowed to transfer in those areas where the above-mentioned arrangement had been carried out. Mr. J. Gillies, the chairman, however, insisted that they could not compel insured persons to remain on a particular panel if they desired to change, however much they all hoped that no transfers would be made from the panels of those doctors who had volunteered for military service. Legally, he must point out, insured persons had a right to change each year if they wished.

### THE THICK END OF THE WEDGE.

Of course, we must all agree that doctors who have gone on active service deserve well not only of their country, but (which, from their point of view, is how the public opinion of their country first expresses itself) of their patients. That is beyond question, and panel patients will assuredly bear it in mind. But Dr. Russell's proposal was extremely mischievous. Panel practice first did away with the promised right of free choice of doctor, by "interpreting" it to mean free choice of *panel* doctor. Now, were Dr. Russell's suggestion acted on, free choice even of panel doctor would be also explained away. Whatever motives may have prompted him, the suggestion itself shows how important it is to preserve elementary rights; for, once they are infringed, as has been the case with the right to free choice of doctor, there is no knowing what oppression may not follow. The above is an example, too remarkable to be passed over, of the thick end of the wedge following on.

### EDUCATION BY EMERGENCY.

The pressure on Poor-Law infirmary accommodation is increasing, and, as the following instance will show, three demands for beds are having to be met at the present time. There is accommodation for Belgian refugees; accommodation for sick among the troops; and accommodation for the ordinary class of case which the winter months on the one hand, and the effects of the war upon the other, are making more numerous than usual. At a recent meeting of the Foleshill Guardians it was suggested that the disused infirmary should be handed over for the accommodation of Belgian refugees, whereupon Dr. Orton remarked that, so far as he knew, the old infirmary was the only building available for even such a purpose as that suggested in a circular received from the Local Government Board, in which the Board asked if there were any accommodation at the Union for any cases of enteric which might occur among the troops. The Guardians themselves were unable to use the old infirmary even for isolating cases, since they had not sufficient nurses. He could not recommend the use of the infirmary, but it might serve in a national emergency. Nurses would have to be obtained; and of course the accommodation would not be offered for nothing. But, on the other hand, their hospital, now full with forty-four cases, could not be offered for a moment, and its use for outside patients was impossible. From this pressure of demand one fact emerges, which is that old and unprogressive methods of Poor-Law administration will have to give way under the coming strain; and the work which the Poor Law will be forced by emergency to do will probably educate Boards of Guardians everywhere throughout the country, as London problems have educated the Metropolitan Asylums Board, so that one of the good results of the war will be the raising the general standard of Poor-Law institutions to the lasting gain of the country as a whole.

### REPATRIATING A MENTAL PATIENT.

The Mental Hospitals Committee of the London County Council have been able to arrange for an alien "pauper lunatic," who since July of last year has been a patient at Long Grove Mental Hospital, to be sent back to the United States. An expulsion order was made by the Home Secretary, and the patient was sent to New York by the *Mauretania* in charge of an attendant. The total cost of the patient's repatriation was £29 15s., and as the Home Office will not defray more than half the cost where the cost of repatriation exceeds £15, the London County Council has just agreed to bear half of the expense—not a bad investment, as it will relieve the county from any further charges for the maintenance of the patient.

### WAR'S EFFECT ON MENTAL HOSPITAL EXPENSES.

Some time ago the London Mental Hospitals Committee, as already announced in The Hospital, intimated that it would probably be necessary to increase the weekly sum charged for the maintenance of patients. Hitherto the charge has been 11s. 1d. a week, but in consequence of the war the prices of supplies have greatly increased and a number of contractors have sent in claims for extra payment for goods which have been supplied since war was declared. These claims are under consideration. No fewer than 360 members of the committee's staff have either been recalled to the Colours or have volunteered for service in the Army or Navy. They are receiving full pay, less the amount allowed to them or their dependants by the Government. The committee have found it absolutely necessary in most instances to engage a temporary staff to fill the place of the absentees. Although economies have been effected in other directions they estimate that the net extra cost of supplies and of temporary staff to the end of next March will amount to some £16,000. They have, therefore, decided to increase the weekly rate to 11s. 8d., an increase of sevenpence a week, which will produce an additional income, it is estimated, of £15,500. At the request of the Guardians the new rate will date from the beginning of October.

### THE CARE OF THE BELGIAN REFUGEES.

The Metropolitan Asylums Board are to be commended for the manner in which they have taken charge of the numerous Belgian refugees, who unfortunately have had to find a shelter in this country. No fewer than 32,013 of these poor people have been received by the Board, and amongst the institutions specially allotted for their residence have been the Alexandra Palace, Earl's Court, Edmonton Refuge, Millfield House, St. Giles' Home, St. Anne's Home, Park Hospital, Hackney Wick Refuge, St. Marylebone Casual Ward, and Poland Street Refuge, the latter being handed over to representatives of the Jewish community. An account of the Belgian refugees at Alexandra Palace was given to The Hospital's Commissioner in the course of a special interview with Dr. H. Cuff, which appeared on September 26, p. 699. They have disposed of the bulk of the Belgians, and had only 6,693 in their institutions on Saturday last. All the refugees have been removed from the Park Hospital, which, in view of the large number of infectious cases in the Metropolis, is to be used again for the accommodation of these patients. Those suffering from ringworm who are at present at the Park Hospital are to be transferred to the Goldie Leigh Homes at Abbey Wood, which the managers have agreed to occupy from the Woolwich Board of Guardians. The use of the Park Hospital for infectious cases will relieve the managers of a great source of anxiety, and from the constant complaints of the numerous Metropolitan authorities that infectious cases in very poor and crowded districts are forced to be nursed at home, to the detriment of the community.

### A FRENCH MENTAL HOSPITAL WAR STORY.

Few of the letters from the Front which have appeared in the columns of lay newspapers have had any special interest for institutional readers, but a marked exception was one by "a motor-cyclist dispatch rider" which appeared in the *Times* of Monday and from which we take the following:—" Evening, sent out with two dispatches, one to officers supposed, by strange coincidence, to be quartered in lunatic asylum." After being mistaken for a spy by an unarmed Army Service Corps man, the writer, arriving at lunatic asylum, " called loudly for concierge, who says officers already left. One out of his crowd of compatriots offers to conduct me to where they now are in the grounds of asylum. Proceed 100 or so yards. Feel convinced that they are in error and turn to return; they press about me more closely; am certain they are lunatics; return to asylum, which has actually been vacated by the officers. Return to gates untouched, but hustled; just before I reach them asylum director appears with lantern, examines me, and apologises for the others mistaking me for spy; assures me there are no lunatics now at asylum; refuse to believe him, but proceed onwards, and eventually find officers required." This is worth reading, not only for its humour, but as showing how difficult it is for the layman, at all events, when placed in an institution to gauge the mental tendencies of its inmates from their behaviour alone.

### A HOUSE OF QUIET.

Attentive readers of the articles which have appeared in The Hospital from medical officers at the Front will not have failed to observe the references made to the nervous shock that is an inevitable outcome of modern methods of warfare. Our writer gave instances of its occurrence on the battlefield, and hospitals at home also have noted it among the wounded. It is as inevitable, this depression of the nervous system, as are the physical wounds by which it is accompanied. The only difference is the practical one, that for patients and wounded suffering from nervous shock no preparations were made. The Chairman of the London Hospital is, therefore, making a useful point in calling public attention to this gap in our preparations for the wounded. " There are a certain number of our gallant soldiers," he points out, " for whom no proper provision is at present obtainable, but is sorely needed. They are men suffering from very severe mental and nervous shock, due to exposure, excessive strain, and tension. They can be cured if only they can receive proper attention. If not cured they will drift back to the world as miserable wrecks for the rest of their lives. A number of physicians have offered their services, free, to attend all these patients if a quiet home in London can be provided and one in the country. The scheme has received the sanction and support of the War Office. If the public will trust me with £10,000, the scheme will be carried through, and I will do my best to see that it is properly worked." It is satisfactory to see that the War Office is now alive to the need for such an institution; these cases for treatment are especially worthy of skilled medical aid in that modern warfare has made them a new class of victim to its weapons.

### THE SECRETARYSHIP OF THE GENERAL HOSPITAL, BIRMINGHAM.

There are certain to be numerous candidates for the chief administrative post at Birmingham General Hospital, which has been rendered vacant through the death of Mr. Howard Collins. The committee apparently have sought to protect themselves against too large a list, since the limitation of age (men not less than thirty, nor more than forty) is more precisely defined than is usual in these advertisements. The salary offered is £500, and it will be interesting to see who is selected on these conditions to fill this highly important and responsible post. Administrative work in Birmingham is both varied and interesting from the high degree of co-ordination which exists between the various branches of charitable work in the city; but this technical advantage also means a great deal of hard and onerous work, which in days like the present deserves and should command an equally high degree of remuneration. The best man can be secured only by such a policy.

### THE AMERICAN MOTOR AMBULANCE CORPS.

As a result of a visit to the French battlefield early in the war, Mr. Richard Norton organised the American Volunteer Motor Ambulance Corps, for which new units are now being raised. Up to the present thirteen ambulances have been dispatched, and the Volunteers are mainly old Harvard and Yale men. The treasurer is Mr. George R. Read, and all funds and offers of Volunteers should be addressed to the Corps, c/o Messrs. Brown, Shipley and Co., 123 Pall Mall, London. The Hon. Secretary is Mr. H. de T. Glazebrook, and the advisory committee includes the distinguished novelist and critic Mr. Henry James. Many more motor ambulances from various sources have been supplied of late, but unfortunately there seems no sign of the supply outstripping the demand.

### THE VINDICATION OF A MEDICAL SUPERINTENDENT.

The Camberwell Board of Guardians have adopted a resolution, with only one dissentient, congratulating Dr. Keats, the medical superintendent of their infirmary, on the result of the three libel actions in which he was involved. The resolution adds that the refutation of the charges of cruelty to children, and by implication against the nursing staff and infirmary committee, was a complete vindication of his character, and they desired to assure him of their continued confidence in him as the chief officer of one of the largest infirmaries in London. The Guardians have also resolved to apply to the Local Government Board for sanction to increase his salary a further £50 a year. The jury in the last case brought by Dr. Keats added a rider to their verdict, which was that "The jury are of opinion that in future all correction of child patients should be reported at the following meeting of the Guardians." The Board in November last decided to provide a punishment book, which is now laid before the infirmary committee at each meeting.

### NOTIFICATION FEES IN PULMONARY TUBERCULOSIS.

Although there is a large amount of money expended every year in the prevention and treatment of consumption, the results taken as a whole are not all that could be wished. But there is one point regarding notification that might be put on a more satisfactory basis without adding appreciably to the expenditure. The general practitioner, with possibly a comfortable income, receives 2s. 6d. for each notification, but the medical officer in a tuberculosis institution, though he has to notify each case on admission and discharge, is entitled to no fee. Previously he was entitled to 1s., which was most acceptable for the extra work, when we consider that the average salary is a modest one. Some public bodies still pay a fee of 1s., though not legally bound to do so; in the case of those that do not pay, the medical officer is out of pocket 2d. in postage for every case coming to an institution. Should medical officers get to know the public bodies who do not pay, is it really reasonable to expect them to notify the cases when it means being out of pocket, to say nothing of the extra work involved? Yet it is undoubtedly most important that all cases should be notified; many cases from time to time change from one district to another, and consequently, if not notified, cannot be kept under the necessary supervision.

### A TREASURY CONCESSION TO MILITARY HOSPITALS.

A generous offer by Councillor P. L. Baker, a well-known Leicester tobacco merchant, has induced the Treasury to make a valuable concession, which will be much appreciated in military hospitals throughout the kingdom. In the early days of the war Mr. Baker offered to present the authorities of the 5th Northern General Hospital with a large quantity of tobacco provided the Excise and Customs authorities freed the gift from duty. The offer was brought by local members of Parliament to the notice of the Treasury, who agreed to make a free weekly allowance of tobacco to troops in the field in France, but regretted they could not extend the concession to men returned sick and wounded to England. Subsequent consideration, however, enabled the Treasury authorities to concede the latter privilege, and instructions were given to Customs and Excise officials to release from bond tobacco in bulk, free from all revenue charges, for the military hospitals. Steps have accordingly been taken to secure Mr. Baker's much-appreciated gift for the soldiers in the Leicester Hospital. The concession will be received with favour by all the military hospitals.

### BRADFORD ROYAL INFIRMARY AND THE CORPORATION SCHEME.

At the quarterly meeting last week of the Bradford Hospital Fund Mr. Herbert Gill expressed the hope that the Local Government Board would shortly sanction the Corporation's scheme for buying the old infirmary's buildings. If the Corporation's scheme were carried out, however, they would have power to appoint a quarter of the board of management, or six members. If that were so, he added, the Hospital Fund Association would have to say how many representatives they would have on the board. "The Fund's contributions," Mr. Gill remarked, "if capitalised, are equal to the contribution which the Corporation would make, according to the scheme which had been put before the Local Government Board." Therefore, if the Corporation were to have six representatives the Hospital Fund ought to have more than two. This speech is worth mentioning, particularly at a time when all our thoughts are mainly bent on one subject, for it shows first, that, as far as may be, ordinary hospital developments are proceeding; and, secondly, it provides but the latest instance of the difficulties of adjusting representation and management, which have been discussed so often in our columns. The one broad fact which emerges out of much detail, varying local conditions and so on, is that, logically, "fair" representation to all parties may easily defeat its own end, by making administration all but impossible through the unwieldiness of committees of management. It must be assumed, in fact, that the committee, whatever be its components, provided that it is not a mere clique, will have the hospital's interests as a whole more at heart than that of any party. Sometimes the reverse is assumed, but experience proves that practical experience on committee is the best education that representatives can have. That is why it is important not to change *personnel* with undue frequency, and how it comes about that theoretical difficulties are solved in practice.

### THE NORTH STAFFORDSHIRE INFIRMARY STAFF.

Since the last account in The Hospital the North Staffordshire Infirmary has lost the services of two of its house surgeons, Mr. R. B. M. Yates, Ch.B. Edin., and Mr. T. A. Peel, M.B., B.S. Durh., both of whom have been called upon for war service. The president of the hospital, Mr. F. J. Harrison, now Lieut.-Commander F. J. Harrison, R.N.R., is at present on active service somewhere upon the sea, in his own steam yacht, doing such work as is entrusted to him. Before leaving home, he generously guaranteed the sum of £1,000 towards the cost of upkeep and of new equipment (about forty-four beds) found necessary if the civilian beds of the hospital were not to be unduly reduced in number while providing for the reception of wounded soldiers. A special fund has also been started in the district to meet these expenses, and is being most generously responded to.

### MENTAL HOSPITAL STAFFS AND WAR LOSSES.

Reference has already been made in The Hospital to the manner in which the staffs of the London mental hospitals have responded to the country's appeals for men to fight its battles. An excellent example has been set them by the members of the Mental Hospitals Committee of the London County Council. Lord Windsor, son of Lord Plymouth, and Major the Hon. A. C. S. Chichester have both found it necessary to resign their membership of the committee owing to the fact that they are on service with the Forces. Claybury Mental Hospital has lost two of its assistant medical officers—Mr. C. St. A. Vivian, the third officer, and Mr. W. Moodie having both resigned to join the Royal Army Medical Corps. We deeply regret to see among the names of those killed in action that of Mr. Sidney Nelson Crowther, senior medical officer at the Surrey County Hospital at Netherene, and formerly assistant medical officer at Brookwood Mental Hospital, and senior house physician at Westminster Hospital, who was serving in the capacity of a motor-cycle dispatch rider at the time he met his death. Mr. Crowther was thirty-nine years of age, and his death will be mourned by a large circle of his friends.

### THE ARCHITECTURE OF EMERGENCY HOSPITALS.

Among the many country seats which are being transformed as emergency hospitals for officers is Balmoral Castle. The fact is interesting in more ways than one, for Balmoral affords an example of that modified Gothic architecture which, for express hospital purposes, has of course been now finally discarded for many years. The reception rooms on the ground floor are, of course, spacious, though even the ballroom is by no means exceptionally large. In the case of the bedrooms a story is told which may be worth repeating. In the active days of John Brown, when Queen Victoria was in the habit of paying her annual visit to Balmoral, the Minister in attendance, whoever he happened to be, was, of course, among the guests who were constantly coming and going. However, not merely was the accommodation limited in itself, but, the story goes, a certain Prime Minister of large physique found difficulty in having his morning bath owing to the smallness of the bedroom. Since then alterations have been made, but it is probably true to say that the nurses who will be installed when hospital work begins in earnest at Balmoral will not find their bedrooms in the castle larger than those of the average nurses' home. The point illustrates one of the chief difficulties of any architectural style based on the Gothic model—namely, that the convenience of the upper rooms is bound to suffer from the limitations which the pointed gable and turret impose. For the Gothic, as Ruskin remarked, is that style which employs the pointed arch for the roof proper and the gable for the roof-mask. Palladian, on the other hand, and we may say all Post-Gothic styles, depend on breadth rather than height, attract the eye horizontally rather than vertically, and the extreme logical development of this principle is the pavilion of modern hospital architecture.

### DISPENSING FEES AND "SWEATING PRICES."

The effect of the new Tariff for drugs and appliances described on p. 106 of last week's Hospital would be to reduce the dispensing fee on a large proportion of medicines prescribed for insured patients to one penny. Considering the low rates of profit on the drugs dispensed, chemists strongly resent this attempt to make use of their professional services at rates which they appear to be justified in describing as "sweating prices," and there is a strong possibility that they will refuse to accept the proposed new terms. But, apart from the matter of dispensing fees, the question arises whether it is in the best interest of the medical profession and of insured persons to encourage the practice of prescribing "stock" mixtures.

### A REMARKABLE COTTAGE HOSPITAL TREASURER.

The voluntary system is distinguished by the long periods of personal service and by the inherited interest of families in institutions, but the forty-two years for which the late Mr. F. R. Willis held the post of honorary treasurer to the Trowbridge Cottage Hospital must almost constitute a record, so far as cottage hospitals are concerned. Ever since the institution was founded in 1870 Mr. Willis has been closely associated with it, and save for the first two years acted as its treasurer throughout his life. No one was better able to pay a tribute to his interest and work than the Rev. P. A. Nash, the president, who at a recent meeting remarked that no one had done more than Mr. Willis for their hospital. Characteristically, for we find this so often in the lives of workers for hospitals, the cottage hospital is by no means the only institution in Trowbridge which has cause to regret Mr. Willis's death. His sympathies were wide, and the practical experience which he acquired in one branch of charitable work he put to interest, as it were, in another. We believe, however, that it was hospital work which first attracted him, and the Trowbridge Cottage Hospital can congratulate itself on having possessed one of the most remarkable of cottage hospital treasurers.

### THE ART OF APPEAL IN WAR TIME.

We have already alluded to the difficulties of hospital finance which have arisen by the war, and later instances show how much the problem of raising money is now exercising the minds of hospital managers. Special appeals are being generally abandoned, but, while this policy prevails, special difficulty is being felt in the case of those institutions which at the beginning of the war were hoping to pay off an accumulated overdraft by means of a special effort. For instance, we find that the Royal Orthopædic Hospital, Birmingham, has been hit in two directions. The question of rebuilding has been before the committee, and some of its chief supporters had been approached to provide a "send off" to an intended appeal. The war has decided them not to proceed with this, but their anxieties, unfortunately, do not end here. Patients and operations have increased in number, and the committee is faced with the need "to arrest the constant increase in the annual deficiency." These deficits have been accumulating for seven years, and legacies alone have saved the situation. The financial crisis at this hospital is, therefore, serious. In some instances, as we hope may be the case with Charing Cross Hospital, the war itself may provide a solution for the difficulties which it has occasioned. At least, what argument more likely to attract public support could be found than that issued in these words by its appeal secretary: "An appeal for £3,000 is made to defray the cost of the five newly-renovated wards for the use of wounded soldiers and sailors. The appeal was about to be made before the war started, but, as time could not be spared to wait for results, the articles necessary for the wards had to be purchased without delay." We trust that this may prove fruitful; but what are hospitals with only an accumulated deficit to plead to do? Have our readers any suggestions?

### TURKEY AND THE DRUG MARKET.

The recent developments in Turkish affairs have naturally drawn attention to the market for opium. Shipments of opium from Turkey during the last few months have been comparatively small, and it now seems as though supplies will be cut off entirely. Fortunately, however, there are unusually large stocks of Persian opium in London, and in consequence there is not likely to be any shortage of morphine or codeine. Up to the time of writing Turkey's intervention in the war had not had a very marked effect on the prices of opium and its alkaloids, but in due course values must almost inevitably rise, although the large available stocks of Persian opium will put a brake on the advances. Generally speaking, the Drug market continues more or less uneventful, the tendency of prices in the case of a fair number of drugs being in a downward direction. In consequence of the decline in the price of citric acid, citrates are considerably cheaper than they were a week or so ago. Spanish ergot is also cheaper. Menthol and eucalyptus oil are offering at prices at which it might pay to buy. Lanoline is much dearer. Liquid paraffin answering the required tests is in small supply. Buchu leaves have a somewhat lower tendency, but senna leaves are dearer.

### TO OUR READERS.

Contributions are specially invited from any of our readers to these columns. They should deal with topical subjects and news. They must be authenticated for the information of the Editor only. The minimum payment if published is 5s. There is no hard-and-fast rule as to space, but notes of about twenty lines in length are preferred.

